# Application of artificial intelligence in screening for adverse perinatal outcomes: A protocol for systematic review

**DOI:** 10.1097/MD.0000000000023681

**Published:** 2020-12-11

**Authors:** Stepan Feduniw, Dorota Sys, Sebastian Kwiatkowski, Anna Kajdy

**Affiliations:** a St. Sophie Specialist Hospital; b Lazarski University, Faculty of Medicine; c Department of Reproductive Health, Centre of Postgraduate Medical Education, Warsaw; d Department of Obstetrics and Gynecology, Pomeranian Medical University, Szczecin, Poland.

**Keywords:** artificial intelligence, artificial neural network, high-risk pregnancies, machine learning, perinatology, pregnancy

## Abstract

The article presents a systematic review protocol. The aim of the study is an assessment of current studies regarding the application of artificial intelligence and neural networks in the screening for adverse perinatal outcomes. We intend to compare the reported efficacy of these methods to improve pregnancy care and outcomes. There are more and more studies that describe the role of machine learning in facilitating the diagnosis of adverse perinatal outcomes, like gestational diabetes or pregnancy hypertension. A systematic review of available literature seems to be crucial to compare the known efficacy and application. Publication of a systematic review in this category would improve the value of future studies. The studies reporting on artificial intelligence application will have a major impact on future prenatal practice.

## 1 Introduction

Prediction of adverse pregnancy outcome (APO) is a challenge of present-day perinatal medicine. Development of prediction models and effective screening methods for APO could improve the diagnostic and therapeutic decision-making process. This could have a significant impact on the wellbeing of both women and children. Routine implementation of artificial intelligence (AI) could even further improve medical care.

AI means the use of computer technology for performing human tasks. The term AI comes from the Czech word “robota,” which means nonhuman work.^[1]^ The history of the development of intelligent machines dates back to 1956. Using the term AI in medicine has 2 primary meanings: virtual and physical. The physical branch of AI is robotic-assisted urologic,^[2]^ and gynecologic^[3]^ surgeries which aim at improving the manual skills of the surgeon. Robotic surgery has found an application also in neurosurgery.^[4]^ On the contrary virtual implementation of AI is used to improve the medical health provider's diagnostic and therapeutic decision-making process.^[4]^

The first constructed robot was based on the human body drawn by Leonardo da Vinci. This model was the inspiration for the physical implementation of the AI and is named DaVinci Surgical Assistance after him (Intuitive Surgical, CA). After the improvement of the operator's manual skills came the idea to improve the mental abilities using deep learning machines and artificial neural networks.^[1,5]^ Artificial neural networks (ANN) is the most popular virtual AI form in medicine.^[5]^ ANNs are computer analytic software inspired by the animal nervous system. ANN is built by interconnected computer processors called “neurons.” These neurons can perform simultaneously complicated data calculations. ANN can learn and analyze imprecise pieces of information, analyze nonlinear data and previous examples. These abilities allow the analysis of medical data and help in diagnostic and treatment decisions.

The artificial intelligence methods have a high potential for application in routine medical practice. It seems that in the future, almost every predictive value of a diagnostic method would be boosted by artificial neural networks.

The scope of this study is the application of virtual learning machines in the assessment of pregnancy risk and prediction of APOs.

We intend to investigate the reported application of these methods to improve pregnancy care and outcomes. There are more and more studies that describe an application of machine learning in screening for high-risk pregnancies and APO, like gestational diabetes or pregnancy hypertension (PH). A systematic review of available literature seems to be crucial to compare known outcomes. Publication of a systematic review in this category would improve the value of future planned and published studies.

## 2 Aim of the study

To investigate the current evidence for the application of artificial intelligence methods in obstetric pregnancy risk assessment and prediction of APOs.

## 3 Review question (Table 1)

- Artificial intelligence methods in the screening for pregnancy risk and APO.

**Table 1:** Review question.

Population	Intervention	Comparison	Outcome
Pregnant women with high-risk pregnancy (with significant complications)	Application of artificial intelligence methods in evaluation of pregnancy risk sand screening for APO	Pregnant women with low-risk pregnancy (with healthy pregnancy)	Prediction value of artificial intelligence methods

## 4 Methods

This study protocol is based on the PRISMA-P guidelines.^[6]^

### 4.1 Study selection

**Figure 1:**
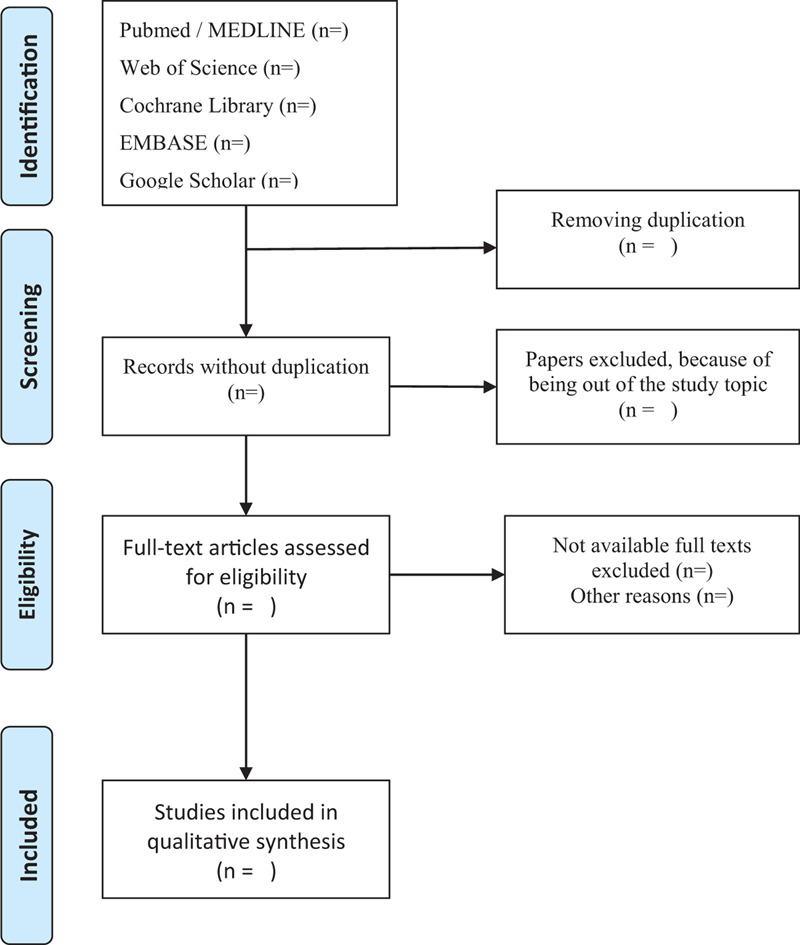
PRISMA flow chart of study selection process.

The process of selecting the eligible literature is shown in a Preferred Reporting Items for Systematic Reviews and Meta-Analyses (PRISMA) flow diagram (Fig. 1)

#### 4.1.1 Searching databases

- Pubmed/MEDLINE,
- Web of Science,
- Cochrane Library,
- EMBASE,
- Google Scholar.

#### 4.1.2 Search strategy

The results would be accessed manually without using any Search software. The general search phrase that will be used is shown in Table 2. Search engine options will be used to limit the search to title and abstract, languages restricted to English, German or Polish, no publication time limits.

**Table 2:** Search strategy.

(pregnant OR pregnancy OR prepartum OR prenatal OR gestation OR prelabour OR maternal) AND (artificial neural networks OR artificial intelligence OR machine learning) AND (pregnancy risk)

### 4.2 Inclusion criteria

#### 4.2.1 Types of studies

All types of evaluative study designs are eligible for inclusion, including grey literature. Studies will not be selected based on methodological quality.

#### 4.2.2 Types of participants

This literature review will compare present evidence for the application of artificial intelligence methods among pregnant women in obstetric risk assessment and prediction of APO.

#### 4.2.3 Types of intervention

Usage of artificial intelligence methods.

#### 4.2.4 Types of exposure

Exposure to APO.

#### 4.2.5 Types of outcome measures

AI could be applied in the assessment of adverse pregnancy outcomes such as gestational diabetes mellitus (GDM), fetal growth restriction (FGR), hypertensive disorder spectrum (pregnancy-induced hypertension, preeclampsia (PE), eclampsia, HELLP syndrome), intrahepatic cholestasis of pregnancy (ICP), fetal structural abnormalities, genetic syndromes (eg, Down, Turner, Edwards, Patau, and others), preterm birth, and other rarer pregnancy complications and prepregnancy complications.

Pregnancies without any complications during pregnancy would be assessed as low-risk. The pregnancies in which any of the listed complications occur would be assessed as high-risk pregnancies.^[7]^

Risk of GDM. GDM diagnosed according to world health organization and the American College of Obstetricians and Gynecologists guidelines.^[8–10]^ The criteria are fasting glycemia 5.1 to 6.9 mmol/L (92–125 mg/dl), first hour of oral glucose tolerance test (75 mg glucose) ≥10 mmol/L (180 mg/dl), or in the second hour of oral glucose tolerance test 8.5 to 11.0 mmol/L (153–199 mg/dl).^[8–10]^

Risk of hypertensive disorder spectrum including PH, PE, eclampsia, and HELLP syndrome. PH diagnosed according to the definition of The American College of Obstetricians and Gynecologists and World Health Organization.^[11,12]^ Diagnosis is made if blood pressure values are ≥140/90 mm Hg, detected for the first time after 20 weeks of pregnancy. The occurrence of proteinuria (≥0.3 g protein in 24-hour urine collection or protein/creatinine ratio ≥0.3) implements the diagnosis of PE.^[13]^

HELLP syndrome is defined, when several symptoms are observed: hemolytic anemia (lactate dehydrogenase ≥600 IU/L and/or bilirubin level in the blood >1.2 mg%), the elevation of the liver enzymes (aspartate transaminase ≥70 IU/L) and thrombocytopaenia (<100 000/ml).^[13]^

Risk of ICP. Government of Western Australia Department of Health and the Royal College of Obstetricians and Gynecologists diagnose ICP as obstetrical pruritus accompanied by an otherwise unexplained elevation in liver function tests or bile acid concentrations, both of which resolve after delivery. The bile acid concentrations >15 mmol/L are diagnostic for ICP.^[14–16]^

Risk of fetal structural abnormalities can be diagnosed in the routine ultrasound examination during pregnancy.^[17]^

Risk of chromosomal abnormalities. Fetuses affected by genetic syndromes (eg, Down, Turner, Edwards, Patau, and others) are suspected on ultrasound and diagnosed by chorionic villus sampling or amniocentesis. However, amniocentesis could be performed in pregnant women in a high-risk group, based on a combination of serum and ultrasound markers.^[18–20]^

Risk of preterm birth. The definition of preterm birth means delivery before 37 weeks of gestation. Preterm delivery results in the highest risk of neonatal complications. A significant time in which the medical support could be implemented is threatening preterm birth. This pregnancy complication occurs when the progression of cervical dilatation (less than 10 points on Bishop scale) is present, and ripening is caused by regular uterine contractions (4–7 contracts per hour) before 37 weeks of pregnancy.^[21,22]^

Risk of FGR. FGR is defined as early and late FGR. Early FGR (<32 weeks) is recognized when abdominal circumference (AC) is <3rd centile, estimated fetal weight (EFW) is <3rd centile, and diastolic flow in the umbilical artery (UA) is absent. Also, early FGR is diagnosed when AC or EFW is <10th centile, and a pulsatility index estimates >95th centile in the UA or uterine artery. Late FGR (≥32 weeks) is diagnosed when AC or EFW are <3rd centile. Also, diagnostic criteria are fulfilled when EFW or AC are <10th centile. Late FHR could also be defined when AC or EFW is higher than 2 quartiles on growth charts, and the cerebroplacental ratio is <5th centile or UA-pulsatility index >95th centile.^[23,24]^ Pathophysiology of FGR is multifactorial, and AI technics could predict this complication occurrence.

Risk of stillbirth. Stillbirth is the fetal death of ≥22 weeks of gestation. Several factors could be used, like illicit drug use, low education state, low socioeconomic status, antenatal care absence, assisted reproductive technology singleton pregnancy, PH, PE, eclampsia, small size for gestational age (<10th centile), post-term pregnancy (≥42 weeks), previous stillbirth.^[25,26]^

#### 4.2.6 Exclusion criteria

Editorials, newspaper articles, and other forms of popular media will be excluded. Failure to meet any one of the above eligibility criteria will result in exclusion from the review, and an independent reviewer will resolve any apparent discrepancies resulting from the selection process. The main reason for exclusion will be using AI methods in another aim that pregnancy risk evaluation. The number of excluded studies (including reasons for exclusion for those excluded following review of the full text) will be recorded at each stage.

### 4.3 Assessment of risk of bias and data extraction

The risk of bias will be assessed independently during the data extraction process by at least two researchers using the Newcastle-Ottawa Scale. The third reviewer will assess any differences. Data on the usage of artificial neural networks in pregnancy risk assessment will be extracted.

Each study will be assessed and compared in several aspects:

- The selection of the study group and the control group
- The comparability of the groups
- The detection of the intervention
- The identification of AI method used
- Knowledge source for AI implementation in the study

A study will reach one star for each signaling question. The questions will be divided into three categories Selection, Comparability, and Exposure/Outcome. Out of 9 possible stars, reaching 7 or more will be evaluated as a high-quality study.

Studies will be divided into 3 categories: low risk of bias, unclear bias, and high risk of bias. The following characteristics will be evaluated:

- Random sequence generation (selection bias)
- Allocation concealment (selection bias)
- Blinding of participants and personnel (performance bias)
- Blinding of outcome assessment (detection bias) (patient-reported outcomes)
- Incomplete outcome data addressed (attrition bias) (Short-term outcomes [2–6 weeks])
- Incomplete outcome data addressed (attrition bias) (Long-term outcomes [>6 weeks])
- Selective reporting (reporting bias)
- Other biases

### 4.4 Heterogeneity and reporting bias

In case of severe methodological, clinical, or statistical heterogeneity pooled results will not be reported. We will identify heterogeneity by both visual inspections of forest plots and statistical methods. Reporting bias will be identified by using funnel plots.

### 4.5 Dissemination and ethics

No ethical approval will be needed because data from previously published studies in which informed consent was obtained by primary investigators will be retrieved and analyzed. A manuscript will be prepared for submission to a peer-reviewed journal. The current protocol was registered at PROSPERO with ID-number: CRD42020199019.

### 4.6 A potential limitation of the study

Different complications will be assessed. Due to this fact, several biases will be present. A potential limitation of the study will be the usage of AI methods on retrospective material, but not its application. Therefore, prospective analysis is required.

## 5 Discussion

AI technology is a progressively developing branch of medicine. There are more and more studies that describe the application of machine learning in screening for high-risk pregnancies and APO, like gestational diabetes or PH, route of delivery, preterm birth, and other complications of pregnant women and fetuses.^[1]^ Many prenatal examinations are used to predict fetal dysfunctions such as aneuploidies by many maternal biochemical markers such as β-human chorionic gonadotrophin (β-hCG) and pregnancy-associated plasma protein-A (PAPP-A).^[18,20]^ Artificial neural networks (ANN) is the most popular virtual AI form in medicine.^[1]^

The artificial intelligence methods have a high potential for application in routine medical practice. It seems that in the future, almost every predictive value of the diagnostic approach could be boosted by artificial neural network usage.^[1]^

Investigation of the reported application of AI methods to improve pregnancy care and its outcomes. A systematic review of available literature seems to be crucial to compare known outcomes. It is all because systematic reviews involve a detailed and comprehensive plan and search strategy derived a priori, with the goal of reducing bias by identifying, appraising, and synthesizing all relevant studies on a particular topic. Reducing biases is the most important factor of choosing systematic review as the main tool in this article, because it helps to eliminate wrong selections of the components on which the article is made. Often, systematic reviews consist of a meta-analysis component which involve usage of statistical techniques to synthesize the data from several studies into a single quantitative estimate or summary effect size. Because of these aspects, our work will be able to help to create the system or model providing analyze pregnant woman and detecting prenatal and fetal disorders.^[27,28]^

Publication of a systematic review in this category would improve the value of future planned and published studies.

## Author contributions

**Conceptualization:** Stepan Feduniw, Dorota Sys, Sebastian Kwiatkowski, Anna Kajdy.

**Investigation:** Stepan Feduniw, Dorota Sys, Sebastian Kwiatkowski, Anna Kajdy.

**Methodology:** Stepan Feduniw, Sebastian Kwiatkowski, Anna Kajdy.

**Project administration:** Stepan Feduniw, Dorota Sys.

**Supervision:** Anna Kajdy.

**Writing – original draft:** Stepan Feduniw, Dorota Sys.

**Writing – review & editing:** Sebastian Kwiatkowski, Anna Kajdy.
